# LRP1 Downregulates the Alzheimer’s β-Secretase BACE1 by Modulating Its Intraneuronal Trafficking^1^,^2^,^3^

**DOI:** 10.1523/ENEURO.0006-15.2015

**Published:** 2015-04-22

**Authors:** Daisuke Tanokashira, Kazumi Motoki, Seiji Minegishi, Ai Hosaka, Naomi Mamada, Akira Tamaoka, Takashi Okada, Madepalli K. Lakshmana, Wataru Araki

**Affiliations:** 1 Department of Demyelinating Disease and Aging, National Institute of Neuroscience, NCNP, Kodaira, Tokyo 187-8502, Japan; 2 Department of Neurology, Faculty of Medicine, University of Tsukuba, Tsukuba, Ibaraki 305-8575, Japan; 3 Department of Biochemistry and Molecular Biology, Nippon Medical School, Bunkyo-ku, Tokyo 113-8602, Japan; 4 Torrey Pines Institute for Molecular Studies, Port St. Lucie, Florida 34987-2352

**Keywords:** Alzheimer’s disease, amyloid beta-protein, BACE1, LRP1, neuron

## Abstract

The β-secretase called BACE1 is a membrane-associated protease that initiates the generation of amyloid β-protein (Aβ), a key event in Alzheimer’s disease (AD). However, the mechanism of intraneuronal regulation of BACE1 is poorly understood. Here, we present evidence that low-density lipoprotein receptor-related protein 1 (LRP1), a multi-functional receptor, has a previously unrecognized function to regulate BACE1 in neurons. We show that deficiency of LRP1 exerts promotive effects on the protein expression and function of BACE1, whereas expression of LRP-L4, a functional LRP1 mini-receptor, specifically decreases BACE1 levels in both human embryonic kidney (HEK) 293 cells and rat primary neurons, leading to reduced Aβ production. Our subsequent analyses further demonstrate that (1) both endogenous and exogenous BACE1 and LRP1 interact with each other and are colocalized in soma and neurites of primary neurons, (2) LRP1 reduces the protein stability and cell-surface expression of BACE1, and (3) LRP1 facilitates the shift in intracellular localization of BACE1 from early to late endosomes, thereby promoting lysosomal degradation. These findings establish that LRP1 specifically downregulates BACE1 by modulating its intraneuronal trafficking and stability through protein interaction and highlight LRP1 as a potential therapeutic target in AD.

## Abstract (precis)

The β-secretase called BACE1 is a membrane-associated protease that initiates the generation of amyloid β-protein, a key event in Alzheimer’s disease. However, the mechanism of inraneuronal regulation of BACE1 is poorly understood.

## Significance Statement

The β-secretase called BACE1 is a membrane-associated protease that initiates the generation of amyloid β-protein, a key event in Alzheimer’s disease. However, the mechanism of intraneuronal regulation of BACE1 is poorly understood. We investigated this issue by focusing on the molecular relationship between BACE1 and low-density lipoprotein receptor-related protein 1 (LRP1), a multifunctional receptor. Our analyses revealed that LRP1 specifically downregulates BACE1 protein expression in human embryonic kidney (HEK) 293 cells and rat primary neurons by facilitating its intracellular trafficking from early to late endosomes through protein interaction, thereby promoting lysosomal degradation. This study thus establishes that LRP1 plays a previously unrecognized role in negatively regulating BACE1 in neurons.

## Introduction

Abnormal accumulation of amyloid β-protein (Aβ) within specific brain regions is thought to play a primary role in the pathogenetic mechanism of Alzheimer’s disease (AD) (Hardy and Selkoe, 2002). Recent evidence also supports the view that soluble Aβ oligomers constitute initiator culprits of AD (Larson and Lesné, 2012). The β-secretase called β-site APP-cleaving enzyme 1 (BACE1) is a membrane-bound aspartyl protease that initiates the generation of Aβ by cleaving the amyloid precursor protein (APP) (Vassar et al., 1999). BACE1 is primarily expressed in neurons in the brain (Vassar et al., 1999) and is possibly involved in AD pathology (Zhao et al., 2007; Vassar et al., 2014). Since BACE1 inhibition is highly effective in reducing Aβ production, BACE1 is an important therapeutic target in AD (Stockley and O’Neill, 2008; Vassar et al., 2014). BACE1 is known to be regulated at transcriptional, post-transcriptional, translational, and post-translational levels (Rossner et al., 2006; Stockley and O’Neill, 2008; Sun et al., 2012), but the mechanisms underlying BACE1 regulation in neurons are only partly understood. Post-translational regulation appears critical, because BACE1 regulation occurs after the protein is matured and transported through neuronal processes.

Low-density lipoprotein receptor-related protein 1 (LRP1) is a transmembrane receptor that belongs to the LDL receptor gene family (Cam and Bu, 2006; Lillis et al., 2008; Zlokovic et al., 2010). LRP1 is a very large molecule (∼600 kDa) that is cleaved by furin to generate a non-covalently associated heterodimer consisting of an α-chain (515 kDa) containing the ligand-binding domains, and a β-chain (85 kDa) containing the transmembrane domain and the cytoplasmic tail (Cam and Bu, 2006; Lillis et al., 2008; Zlokovic et al., 2010). LRP1 is highly expressed in the brain and exerts multiple functions, including endocytosis of specific proteins. Many ligands for LRP1 exist, including apolipoprotein E and α2-macroglobulin, which are also risk factors for AD (Ulery and Strickland, 2000; Cam and Bu, 2006; Lillis et al., 2008; Zlokovic et al., 2010). LRP1 is known to act through scaffolding proteins such as Fe65 to play a role in the endocytosis of APP (Wagner and Pietrzik, 2012), and possibly serves as a substrate of BACE1 (von Arnim et al., 2005). Interestingly, the LRP1 intracellular domain has been reported to be important for targeting APP and BACE1 to lipid rafts (Yoon et al., 2007), considered important sites for the generation and accumulation of Aβ (Vetrivel and Thinakaran 2010; Hicks et al., 2012). Thus, LRP1 may have a role in Aβ production, possibly through interaction with APP and/or BACE1; however, the exact relationship between LRP1 and BACE1 remains unclear. LRP1 is also known to play active roles in Aβ clearance such that it mediates brain-to-blood Aβ clearance at the blood−brain barrier (Cam and Bu, 2006; Lillis et al., 2008; Zlokovic et al., 2010; Kanekiyo and Bu, 2014). In the current study, we focused on the molecular relationship between BACE1 and LRP1, and discovered that LRP1 downregulates BACE1 by modulating its intracellular trafficking and stability through protein interaction in neurons.

## Materials and Methods

### Cell culture

The mouse embryonic fibroblast lines, MEF-1 (LRP1-wild-type (WT)) and PEA-13 (LRP1-knockout (KO)), obtained from American Type Culture Collection, and human embryonic kidney (HEK) 293 cells were cultured in DMEM supplemented with 10% fetal bovine serum. HEK293 cells were cultured on collagen I-coated dishes or plates (Iwaki). Primary neuronal cultures were prepared from cerebral cortices of rat embryos at embryonic day 17, as described previously (Araki et al., 2001; Motoki et al., 2012). Cells were plated on poly-L-lysine–coated dishes or plates and maintained in Neurobasal medium containing B27 supplements (Invitrogen).

### cDNA transfection and recombinant adenovirus infection

HEK293 cells on a six-well plate were transfected with appropriate cDNAs using Lipofectamine 2000 (Invitrogen) according to manufacturer’s instructions. The cDNA constructs used were BACE1 and BACE2 with a C-terminal rhodopsin (rho) tag (generous gifts from Dr. Michael Farzan, The Scripps Research Institute, Jupiter, FL) (Farzan et al., 2000), LRP-L4 (Takeda et al., 2003) (see Fig. 2*A*), myc-tagged dynamin K44A (a generous gift from Dr. Mark A. McNiven, Mayo Clinic, Rochester, MN), and WT APP695 (Takeda et al., 2004). All cDNAs were subcloned into the pcDNA3.1 vector (Invitrogen). Recombinant adenoviruses expressing rho-tagged BACE1, WT APP, Swedish mutant APP695 (swAPP) (Motoki et al., 2012), LacZ (Araki et al., 2001), or LRP-L4 were prepared using an Adenovirus Dual Expression Vector Kit (Takara Bio) according to manufacturer’s instructions. Rat primary cultured neurons were infected with each recombinant adenovirus at a multiplicity of infection (moi) of 5 at 7-8 d *in vitro*.

### Antibodies

The antibodies used were as follows: anti-BACE1 [AB5832, Millipore; D10E5, Cell Signaling; MAB9311, R & D systems; and NBA (Murayama et al., 2005)]; anti-LRP1 1704 (Pietrzik et al., 2002); anti-APP R37 (Kametani et al., 1993); anti-rhodopsin tag 1D4 (University of British Columbia) (Farzan et al., 2000; Murayama et al., 2005); anti-β-galactosidase (LacZ; MP Biomedicals); anti-β-actin (Sigma); anti-myc (Invitrogen); anti-hemagglutinin (anti-HA; rabbit: MBL; goat: Abcam); anti-flotillin-1 (IBL); anti-EEA1 (rabbit: Affinity BioReagents; goat: Biorbyt); anti-γ1-adaptin (Santa Cruz Biotechnology); anti-β-COP (Thermo Scientific); anti-rab7a (rabbit: Millipore); anti-rab7 (mouse: Abcam); and anti-GM130 (BD Biosciences).

### Western blot analysis

Cells were lysed in radioimmunoprecipitation assay (RIPA) buffer containing protease inhibitors. Western blotting of cell lysates was performed using a standard procedure as described previously (Murayama et al., 2006). Protein band densities were quantified using an image analyzer LAS-1000 (Fuji Film).

### Coimmunoprecipitation

Membrane proteins were extracted from HEK293 cells coexpressing BACE1 and LRP-L4 and immunoprecipitated with 1D4 antibody as described previously (Murayama et al., 2006). Immunoprecipitated proteins were analyzed by Western blotting with anti-LRP1 antibody. Anti-BACE1 (MAB9311) was used for immunoprecipitation in coimmunoprecipitation experiments of endogenous BACE1 and LRP1.

### Aβ measurement

The amounts of Aβ40 in conditioned media were measured using sandwich ELISA kits (Wako), as described previously (Motoki et al., 2012).

### Immunocytochemistry

HEK293 cells or primary neurons cultured on cover slips were fixed with 4% paraformaldehyde in PBS. Fixed cells were permeabilized and blocked with 0.3% Triton X-100 and 1% horse serum in PBS, and incubated with primary antibody for 1 h, followed by incubation with Alexa488-conjugated anti-mouse or anti-rabbit IgG secondary antibody (Molecular Probes) for 1 h. For double immunolabeling, cells were subsequently stained with a second primary antibody, followed by incubation with Alexa568-conjugated anti-goat or anti-mouse IgG, Cy5-conjugated anti-mouse IgG, or DyLight649-conjugated anti-rabbit IgG secondary antibody (Jackson ImmunoResearch Laboratories), as appropriate. For triple immunofluorescence staining with antibodies against rhodopsin (1D4) and HA tags, and an antibody against organelle markers (EEA1, rab7a, β-COP, or γ1-adaptin), cells were first incubated with primary antibodies against the organelle marker followed by incubation with the appropriate secondary antibody, then with goat anti-HA followed by incubation with Alexa568-conjugated anti-goat IgG, and finally with 1D4 followed by incubation with Cy5-conjugated anti-mouse IgG. For triple immunostaining of neurons, goat anti-EEA1 and rabbit anti-HA antibodies were used. In some cases, CanGet Signal Immunostain Immunoreaction Enhancer Solution (Toyobo) was used to increase the sensitivity of the reaction with primary antibodies.

For triple-immunofluorescence staining of neurons with antibodies against BACE1, LRP1, and organelle markers (EEA1, Rab7, GM130), cells were incubated with anti-LRP1 followed by incubation with Dylight649-conjugated anti-rabbit IgG, then with anti-BACE1 (D10E5) prelabeled with Alexa568 by Zenon Rabbit IgG Labeling Kits (Molecular Probes), and finally with anti-EEA1, anti-Rab7, or anti-GM130 followed by incubation with Alexa488-conjugated anti-goat or anti-mouse IgG.

### Lipid raft isolation

Lipid rafts were isolated using sucrose density gradient ultracentrifugation, as described previously (Motoki et al., 2012). Generally, the lipid raft marker flotillin-1 fractionated into fraction 4 of the 10 fractions collected.

### Cell-surface biotinylation

Cell-surface biotinylation was performed using a Sulfo-NHS-LC-Biotinylation Kit (Pierce), essentially as described previously (Murayama et al., 2005).

### Cycloheximide chase experiments

Cells were plated on six-well plates, incubated in the presence of cycloheximide (100 μM) for up to 12 h, and analyzed by Western blotting, as described previously (Araki et al., 2006). In cotreatment experiments, cells were coincubated with cycloheximide and chloroquine (50 μM).

### Statistics

All results are presented as means ± SEMs. Data were statistically analyzed using one-way ANOVA followed by a Tukey multiple comparison test or Student’s *t* test with a significance threshold of *p* < 0.05.

## Results

### BACE1 protein expression is increased in LRP1-knockout cells

**Figure 1:**
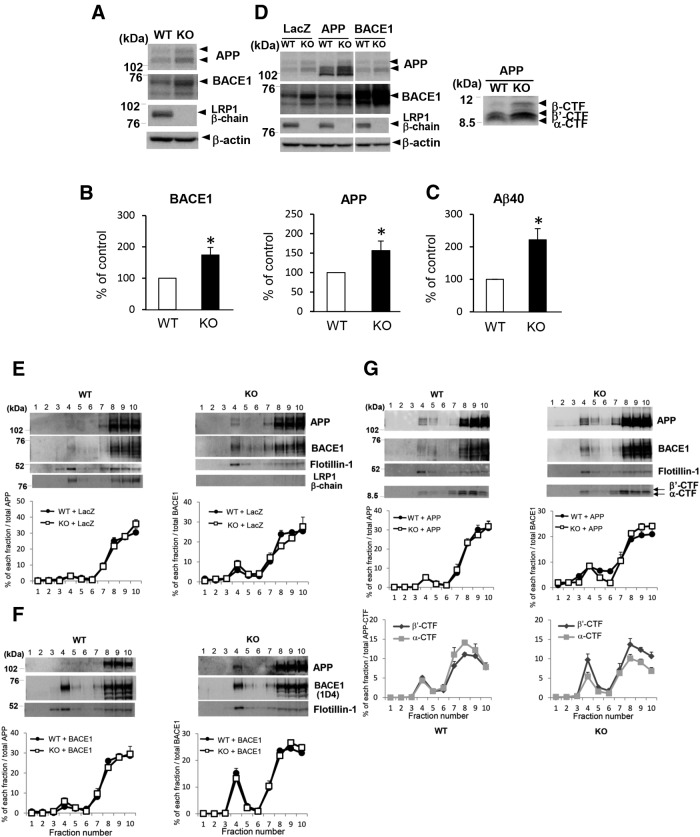
BACE1 protein expression is increased in LRP1-KO cells relative to WT cells. ***A***, Cell lysates of LRP1-KO and WT cells were analyzed by Western blotting with antibodies against APP, BACE1, and LRP1. ***B***, Quantification of relative protein levels in ***A*** (*n* = 3, *p < 0.05). ***C***, The amounts of Aβ40 in 24-h-conditioned media from LRP1-KO and WT cells were measured by sandwich ELISA (*n* = 3, *p < 0.05). ***D***, LRP1-KO and WT cells infected with recombinant adenoviruses expressing LacZ, WT APP, or rho-tagged BACE1 were maintained for 2 d, and cell lysates were analyzed by Western blotting as above. APP CTFs were analyzed by immunoprecipitation−Western blot analysis with anti-APP antibody, as described previously (Motoki et al., 2012). Images from the same blots were merged in the left panel. ***E−G***, LRP1-KO and WT cells infected with recombinant adenoviruses expressing LacZ (***E***), BACE1 (***F***), or WT APP (***G***) were subjected to sucrose density gradient fractionation, as described in Materials and Methods. Each fraction was analyzed by Western blotting with antibodies against the rhodopsin tag (1D4), APP, BACE1, or flotillin-1. The level of BACE1 or APP in each fraction was quantified and expressed as a percentage of the total level in all fractions. For detection of APP CTFs in ***G***, each fraction was additionally analyzed by immunoprecipitation−Western blot analysis with an anti-APP antibody, and the levels of α-CTF and β’-CTF in each fraction were quantified as above (*n* = 3).

We first compared the protein expression level of BACE1 in WT and LRP1-KO cells. Western blot analyses of cell lysates showed that the protein expression levels of endogenous BACE1 and APP in LRP1-KO cells were significantly higher than those in WT cells (Fig. 1*A*,*B*). The increase in APP level in LRP1-KO cells is consistent with a previous report (Liu et al., 2007). The specificity of anti-BACE1 antibody (AB5832) was confirmed by comparison with another anti-BACE1 antibody (D10E5), as presented below (see Fig. 3*E*). Additionally, sandwich ELISAs revealed that the amount of Aβ40 in conditioned media of LRP1-KO cells was higher than that in media conditioned by LRP1-WT cells (Fig. 1*C*). Overexpression of BACE1 or APP in LRP1-KO and LRP1-WT cells using recombinant adenoviruses resulted in much higher levels of BACE1 or APP in LRP1-KO than in LRP1-WT cells (Fig. 1*D*).

It was previously reported that exogenously expressed intracellular domain of LRP1 facilitates lipid raft targeting of BACE1 and APP (Yoon et al., 2007). We compared the lipid raft distribution of endogenous and overexpressed BACE1 and APP between LRP1-KO and LRP1-WT cells by sucrose density gradient fractionation. Although the levels of APP or BACE1 in raft (mainly fraction 4) and non-raft (fractions 7-10) fractions appeared relatively higher in LRP1-KO than LPR1-WT cells in the blots, there were no appreciable differences in the distribution pattern of endogenous (Fig. 1*E*) or exogenous BACE1 (Fig. 1*F*) or APP (Fig. 1*G*), as assessed by the relative level in each fraction of the total level, between the two cell types. These findings suggest that endogenous LRP1 does not affect lipid raft association of BACE1 or APP. Interestingly, consistent with an increased levels of Aβ40 in LRP1-KO cells, an analysis of APP C-terminal fragments (CTFs) indicated that β’-CTF, a metabolite derived from β-secretase processing of APP (Zhou et al., 2011; Motoki et al., 2012), predominated in LRP1-KO cells compared with α-CTF, a product of α-secretase processing of APP, whereas α-CTF was more prominent than β’-CTF in LRP1-WT cells (Fig. 1*D*,*G*), implying that APP processing by BACE1 is promoted in LRP1-KO cells. These data suggest that the steady-state levels, but not lipid raft association, of BACE1 may be regulated by LRP1.

### LRP1 downregulates BACE1 protein expression in HEK293 cells and primary neurons

**Figure 2:**
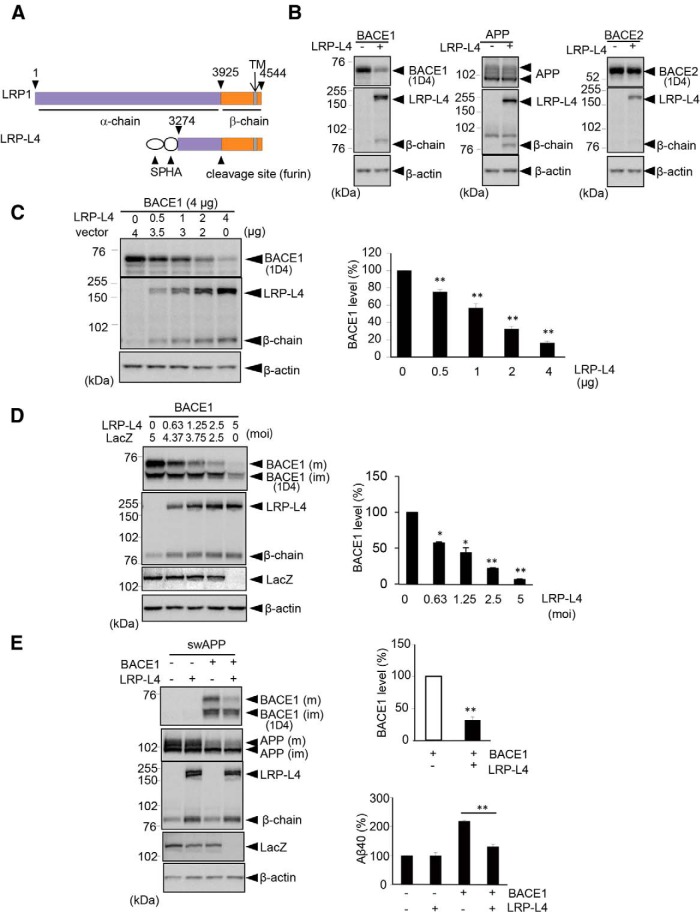
LRP-L4 down-regulates BACE1 in HEK293 cells and primary neurons. ***A***, LRP1 constructs. LRP-L4 is a functional LRP1 mini-receptor; an HA epitope was inserted into LRP-L4 after the signal peptide (SP) at the N-terminus. LRP1 is cleaved by furin to generate an N-terminal α-chain and a C-terminal β-chain. TM, Transmembrane domain. ***B***, HEK293 cells were cotransfected with BACE1 and either LRP-L4 or empty vector. Similarly, cells were cotransfected with APP and LRP-L4 or BACE2 and LRP-L4. Cell lysates were analyzed by Western blotting with the indicated antibodies. ***C***, HEK293 cells were cotransfected with BACE1 and the indicated amounts of LRP-L4 and/or empty vector. Cell lysates were analyzed by Western blotting with 1D4 or anti-LRP1 antibody. Relative BACE1 levels were quantified and graphed. ***D***, Primary cultured neurons were infected with adenoviruses expressing BACE1 (5 moi) plus those expressing the indicated amounts of recombinant LRP-L4 and/or LacZ, and cell lysates were analyzed by Western blotting after 2 d. The graph indicates relative mature BACE1 levels. M, Mature; im, immature. ***E***, Primary neurons were coinfected with the indicated adenoviruses expressing swAPP, BACE1, and/or LRP-L4 (3 moi each), and maintained for 2 d. The total amount of adenovirus infected was equalized by addition of LacZ adenovirus. Cell lysates were analyzed by Western (*continued in page 6*). blotting as above. The graph indicates relative mature BACE1 levels. Aβ40 levels in 24-h-conditioned media were determined with sandwich ELISA. ***C−E***, *n* = 3; *p < 0.05, **p < 0.01.

Having confirmed an essential role of endogenous LRP1 in regulating BACE1 levels, we next used LRP-L4, a functional LRP1 mini-receptor containing an N-terminal HA tag (Fig. 2*A*), to investigate whether exogenously expressed LRP1 can influence BACE1. Coexpression of BACE1 with the C-terminal rhodopsin (rho) tag and LRP-L4 in HEK293 cells induced a remarkable decrease in the level of BACE1 protein compared with that in cells expressing BACE1 alone (Fig. 2*B*), an effect that was dependent on the expression level of LRP-L4 (Fig. 2*C*). In contrast, the levels of BACE2 and APP proteins were not affected by LRP-L4 (Fig. 2*B*), suggesting a specific effect of LRP-L4 on BACE1. Similarly, primary cultured neurons coexpressing rho-tagged BACE1 and LRP-L4 via recombinant adenoviruses showed reduced levels of BACE1 compared with neurons expressing BACE1 and LacZ. Again, this effect was found to be dependent on the expression level of LRP-L4, as indicated by a clear inverse relationship between the amount of LRP-L4 and BACE1 levels (Fig. 2*D*). These data suggest that LRP1 negatively regulates BACE1 protein expression in both HEK293 cells and primary cultured neurons.

Furthermore, primary cultured neurons coexpressing swAPP, BACE1, and LRP-L4 secreted lower amounts of Aβ40 than those coexpressing swAPP, BACE1, and LacZ; this effect was attributable to the remarkable reduction in BACE1 levels (Fig. 2*E*). In contrast, LRP-L4 and β-chain levels were comparable between neurons coexpressing BACE1 and swAPP and those expressing swAPP only. In addition, the levels of both cellular APP and secreted Aβ40 were unaltered in neurons coexpressing swAPP plus LRP-L4 compared with those expressing swAPP only (Fig. 2*E*). These data suggest that LRP1 may inhibit Aβ production through downregulation of BACE1 in primary cultured neurons.

### Physical interaction and colocalization of BACE1 and LRP1

**Figure 3:**
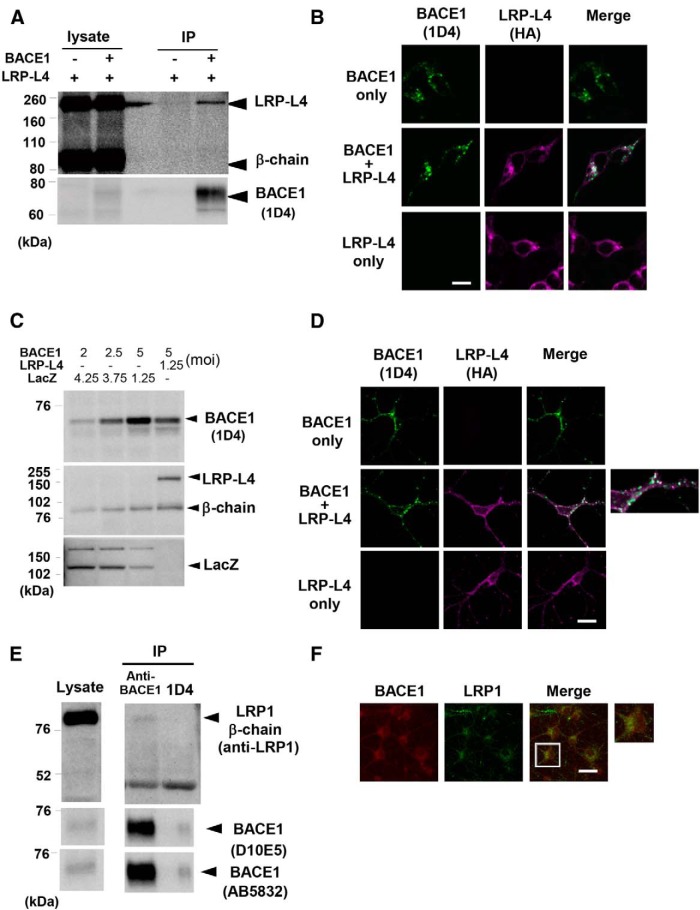
Physical association and colocalization of BACE1 and LRP-L4. ***A***, HEK293 cells were transfected with LRP-L4 and either BACE1 or empty vector. Protein extracts of membrane fractions were immunoprecipitated with 1D4 antibody and the precipitated proteins were analyzed by Western blotting, as described in Materials and Methods. ***B***, HEK293 cells transfected with BACE1 plus LRP-L4, BACE1 only, or LRP-L4 only were analyzed by double-immunofluorescence staining with 1D4 (green) and anti-HA (magenta) antibodies. Overlapping 1D4 and HA immunoreactive signals were observed in cells coexpressing BACE1 and LRP-L4. Scale bar, 10 μm. ***C***, Primary neurons were infected with the indicated amounts of recombinant adenoviruses expressing BACE1, LRP-L4, and/or LacZ. Two days after infection, cell lysates were analyzed by Western blotting with 1D4, anti-LRP1, or anti-β-galactosidase. Comparable BACE1 levels were observed in neurons infected with BACE1 adenoviruses (2.5 moi) only and those infected with BACE1 (5 moi) and LRP-L4 (1.25 moi) adenoviruses. ***D***, Primary neurons grown on coverslips were infected with BACE1 plus LacZ adenoviruses, BACE1 plus LRP-L4 adenoviruses, or LRP-L4 plus LacZ adenoviruses. Cells were analyzed as in ***B***. Neurons coexpressing BACE1 and LRP-L4 exhibited overlapping 1D4 and HA immunoreactive signals in soma and (*continued in page 8*). neurites. Scale bar, 20 μm. ***E***, Protein extracts of membrane fractions of primary neurons were immunoprecipiated with anti-BACE1 (MAB9311) or 1D4 (negative control), followed by immunoblotting with anti-LRP1. The blots were reprobed with anti-BACE1 antibodies (AB5832 and D10E5). Images from the same blots were merged in this figure. ***F***, Primary neurons grown on coverslips were analyzed by double-immunofluorescence staining with anti-LRP1 and Alexa568-labeled anti-BACE1. BACE1 and LRP1 immunoreactivities were clearly overlapped in both soma and neurites of neurons. Scale bar, 20 μm.

We next investigated the mechanism by which LRP-L4 downregulates BACE1. We analyzed interactions between the two molecules by coimmunoprecipitation, using HEK293 cells transfected with BACE1 and LRP-L4 or LRP-L4 alone. These experiments revealed that LRP-L4 was coimmunoprecipitated with BACE1, suggesting that the BACE1 interacts with LRP-L4 (Fig. 3*A*). Notably, full-length LRP-L4 rather than the β-chain primarily immunoprecipitated with BACE1, implying that the N-terminal extracellular region of LRP-L4 is important for the interaction.

**Figure 4:**
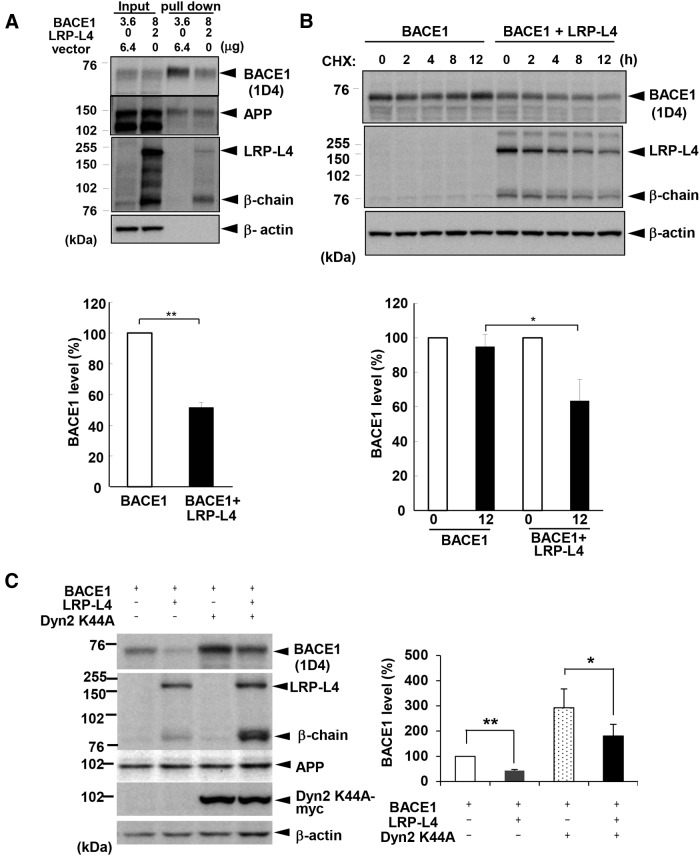
LRP1 decreases BACE1 stability and reduces the cell surface expression of BACE1. ***A***, HEK293 cells were transfected with the indicated amounts of BACE1 and either LRP-L4 or vector. Cell-surface biotinylation experiments were performed as described in Materials and Methods. Western blots of total cell lysates and avidin-agarose-precipitated material are shown. Relative BACE1 levels were quantified and graphed. ***B***, HEK293 cells transfected with BACE1 plus LRP-L4 or vector as in ***A*** were subjected to cycloheximide chase experiments, as described in Materials and Methods. After incubation with cycloheximide (CHX) for the indicated times, cells were lysed and analyzed by Western blotting. Relative BACE1 levels at 0 and 12 h were quantified and graphed. (***A***, ***B***: *n* = 3, *p < 0.05, **p < 0.01). ***C***, HEK293 cells were cotransfected with BACE1, LRP-L4, and/or Dyn2 K44A as indicated. The total amount of DNA was equalized by the addition of vector. Cell lysates were analyzed by Western blotting with appropriate antibodies. Relative BACE1 levels in blots were quantified and graphed (*n* = 3, *p < 0.05, **p < 0.01).

We then performed immunocytochemical analyses to examine whether LRP-L4 influences the intracellular localization of BACE1 in HEK293 cells and primary neurons. In these experiments, comparable levels of BACE1 were expressed in cells coexpressing BACE1 and LRP-L4 and those expressing BACE1 alone by adjusting the amounts of BACE1 cDNA or recombinant BACE1 adenoviruses used for transfection or transduction, respectively (Figs. 3*C*, 4*A*). BACE1 and LRP-L4 were visualized using antibodies to the rhodopsin tag (1D4) and hemagglutinin (HA) tag, respectively. Immunostaining for 1D4 revealed positive granular staining in the cytosol in BACE1-expressing HEK293 cells. Interestingly, the size of 1D4-positive granules appeared to be larger in cells coexpressing BACE1 and LRP-L4 compared to those expressing BACE1 alone (Fig. 3*B*). Double-immunofluorescence staining with anti-1D4 and anti-HA antibodies revealed positive HA immunostaining in the cytosol and nuclear membrane, and showed that HA immunoreactivity partially overlapped with that of 1D4, indicating colocalization of BACE1 and LRP-L4 (Fig. 3*B*). The HA-immunostaining pattern was comparable between cells expressing BACE1 alone and those expressing BACE1 and LRP-L4. Similar findings were obtained with primary neurons expressing BACE1 and/or LRP-L4 as well. Immunostaining for 1D4 showed that immunopositive granules localized in perikarya and neuritic processes. Immunostaining for HA showed positive reticular immunoreactivity in perikarya and neurites. In neurons coexpressing BACE1 and LRP-L4, 1D4 and HA immunoreactive signals partially overlapped, indicating colocalization of BACE1 and LRP-L4 (Fig. 3*D*).

Furthermore, coimmunoprecipitation experiments showed that endogenous LRP1 β-chain was coprecipitated with BACE1 by anti-BACE1 antibody, but not by negative control antibody (Fig. 3*E*), suggesting that endogenous BACE1 and LRP1 interact physically with each other. Since native LRP1 α-chain and β-chain are connected through noncovalent interaction, it is possible that BACE1 physically associated with such a native form of LRP1. Double immunofluorescence staining using Alexa568-labeled anti-BACE1 antibody also clearly showed that the immunoreactivities of endogenous BACE1 and LRP1 overlapped in soma and neurites, demonstrating their colocalization in these subcellular regions (Fig. 3*F*).

### LRP-L4 decreases BACE1 stability and reduces the cell-surface expression of BACE1

To gain a more in-depth understanding of the inhibitory effect of LRP-L4 on BACE1, we performed cell-surface biotinylation experiments in which biotinylated proteins were avidin-agarose precipitated and subjected to Western blotting. In this experiment, levels of BACE1 in cells expressing BACE1 only were comparable to those in cells expressing BACE1 plus LRP-L4. The level of mature, cell-surface BACE1 in cells expressing BACE1 plus LRP-L4 was significantly lower than that in cells expressing BACE1 only (Fig. 4*A*). This finding suggests that LRP-L4 affects BACE1 protein levels at the plasma membrane, possibly by altering BACE1 intracellular trafficking.

We surmised that LRP-L4 might affect the stability of BACE1 proteins, and therefore performed cycloheximide chase experiments. During the 12 h chase period, BACE1 levels were only slightly reduced in cells expressing BACE1 only, whereas the levels were markedly reduced (to ∼60% of the control level) in cells expressing BACE1 plus LRP-L4 (Fig. 4*B*). These data suggest that LRP-L4 decreases the stability of BACE1.

To further examine whether BACE1 downregulation by LRP-L4 is associated with endocytosis from the cell surface, we coexpressed mutant dynamin 2 (Dyn2 K44A), which inhibits endocytosis (McNiven et al., 2000), with BACE1 and LRP-L4. LRP-L4 caused downregulation of BACE1 even with endocytosis inhibited by expression of myc-tagged Dyn2 K44A (Fig. 4*C*). The protein level of BACE1 as well as that of LRP-L4, especially the β-chain, was higher in cells transfected with Dyn2 K44A than in cells without Dyn2 K44A, suggesting that inhibition of endocytosis results in the accumulation of both proteins. These data suggest that downregulation of BACE1 caused by LRP-L4 may be independent of endocytosis.

### LRP1 facilitates BACE1 transport from early to late endosomes, promoting BACE1 lysosomal degradation

**Figure 5:**
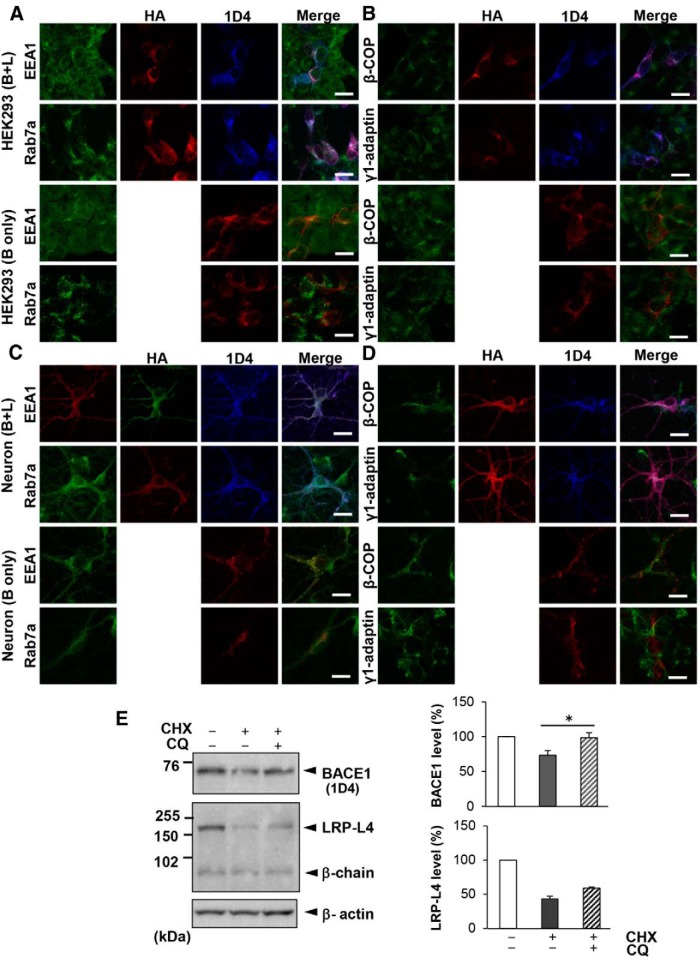
LRP1 induces a shift in the subcellular localization of BACE1 from early to late endosomes in both HEK293 and primary neurons, likely promoting lysosomal degradation. ***A***, HEK293 cells transfected with BACE1 plus LRP-L4 (B+L) or BACE1 only (B only) were analyzed by triple-immunofluorescence staining with anti-EEA1/anti-rab7a (green), anti-HA (red), and 1D4 (blue) antibodies, or double-immunofluorescence staining with anti-EEA1/anti-rab7a (green) and 1D4 (red) antibodies. Note that colocalization of HA, 1D4, and EEA1/rab7a immunoreactive signals was observed in cells expressing BACE1 and LRP-L4, whereas colocalization of signals for 1D4 and EEA1, but not rab7a, was observed in cells expressing BACE1 only. Scale bars, 20 μm. ***B***, HEK293 cells transfected with BACE1 plus LRP-L4 or BACE1 only were analyzed by triple-immunofluorescence staining with anti-β-COP/anti-γ1-adaptin (green), anti-HA (red), and 1D4 (blue) antibodies, or double-immunofluorescence staining with anti-β-COP/anti-γ1- (*continued in page 11*). adaptin (green) and 1D4 (red) antibodies, as in ***A***. ***C***, Primary neurons grown on coverslips were infected with BACE1 plus LRP-L4 adenoviruses or BACE1 plus LacZ adenoviruses. Cells were analyzed as in ***A***, except that goat anti-EEA1 and rabbit anti-HA antibodies were used. Neurons coexpressing BACE1 and LRP-L4 exhibited overlapping 1D4, HA, and EEA1/rab7a immunoreactive signals in soma and neurites, whereas neurons expressing only BACE1 exhibiting overlapping signals of 1D4 and EEA1, but not rab7a. Scale bars, 20 μm. ***D***, Primary neurons infected with BACE1 plus LRP-L4 adenoviruses or BACE1 plus LacZ adenoviruses were analyzed by triple- or double-immunofluorescence analysis performed with anti-β-COP/anti-γ1-adaptin, anti-HA, and 1D4 antibodies, as in ***B***. Scale bars, 20 μm. ***E***, HEK293 cells transfected with BACE1 plus LRP-L4 were subjected to cycloheximide (CHX) chase experiments, in which cells were coincubated with or without chloroquine (CQ; 50 μM). After 12 h, cells were lysed and analyzed by Western blotting. Relative levels of BACE1 and LRP-L4 were quantified and graphed (*n* = 3, *p < 0.05).

We hypothesized that LRP-L4 may alter BACE1 subcellular localization to promote its degradation, and therefore investigated the subcellular location of BACE1 using HEK293 cells and primary neurons. To this end, we performed triple immunostaining with anti-HA, 1D4, and antibodies against early endosomal antigen 1 (EEA1), rab7a, β-COP, or γ1-adaptin, markers of early endosomes, late endosomes, the Golgi apparatus, and the *trans*-Golgi network (TGN), respectively. In HEK293 cells coexpressing BACE1 and LRP-L4, 1D4 and HA immunoreactive signals partially overlapped with those of EEA1 and rab7a (Fig. 5*A*). In contrast, in HEK293 cells expressing only BACE1, 1D4 immunoreactivity clearly overlapped with that of EEA1, but only marginally with that of rab7a (Fig. 5*A*). We additionally observed only limited colocalization of 1D4 immunoreactive signals with those of β-COP or γ1-adaptin in both cells expressing only BACE1 and those expressing BACE1 and LRP-L4 (Fig. 5*B*). The finding that 1D4-positive granules in cells coexpressing BACE1 and LRP-L4 were larger than those in cells expressing only BACE1 seemed to reflect the different subcellular locations of BACE1 in these cells.

Moreover, 1D4 and HA immunoreactivity colocalized with that of EEA1 and rab7a in both soma and neurites of primary neurons coexpressing BACE1 and LRP-L4, whereas 1D4 was substantially colocalized with EEA1, but not with rab7a, in neurons expressing BACE1 only (Fig. 5*C*). Colocalization of 1D4, HA, and β-COP or γ1-adaptin immunoreactive signals was marginal in neurons coexpressing BACE1 and LRP-L4 as well as those expressing BACE1 only (Fig. 5*D*). The presence of EEA1 and rab7a immunoreactivity in neuronal processes is consistent with the notion that endosomal organelles are distributed throughout the soma, dendrites, and axons (Lasiecka and Winckler, 2011). These data suggest that LRP1 facilitates BACE1 sorting from early to late endosomes in both HEK293 cells and primary neurons.

We reasoned that LRP-L4 promotes trafficking of BACE1 to lysosomes, where BACE1 degradation occurs. To clarify this point, we tested the effect of chloroquine, a lysosomotropic agent, in cycloheximide chase experiments, as described above. Treatment of HEK293 cells coexpressing BACE1 and LRP-L4 with cycloheximide plus chloroquine almost completely rescued the reduction in BACE1 levels in cells treated with cycloheximide only, suggesting that LRP-L4 likely destabilizes BACE1 through increased lysosomal degradation (Fig. 5*E*). In addition, the reduced level of full-length LRP1 in cycloheximide-treated cells was only partially recovered by cotreatment with cycloheximide and chloroquine (Fig. 5*E*).

**Figure 6:**
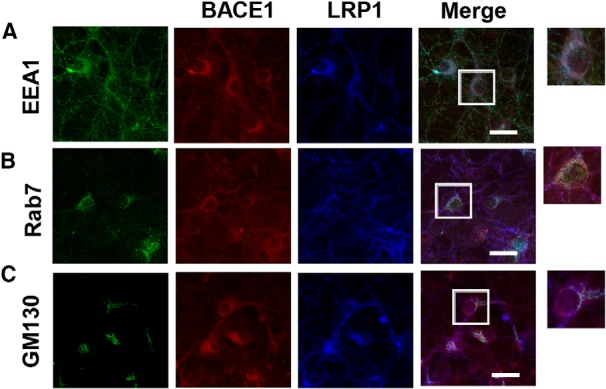
Subcellular localization of endogenous BACE1 and LRP1 in neurons. ***A***, Primary neurons grown on coverslips were analyzed by triple-immunofluorescence staining with anti-BACE1 (red), anti-LRP1 (blue), and anti-EEA1 (green), as described in Materials and Methods. Overlapping immunoreactivities were significantly observed in both soma and neurites of neurons. ***B***, Triple-immunofluorescence staining with anti-BACE1, anti-LRP1, and anti-rab7 exhibited overlapping immunoreactivities mainly in soma of neurons. ***C***, Triple-immunofluorescence staining with anti-BACE1, anti-LRP1, and anti-GM130 displayed only limited overlapping immunoreactivities in soma of neurons. Scale bars, 20 μm.

Finally, we used triple immunofluorescence staining to analyze the subcellular localization of endogenous BACE1 and LRP1 in neurons. The results revealed that both endogenous BACE1 and LRP1 immmunoreactivities were clearly colocalized with EEA1 in soma and neurites and partially with rab7 mainly in soma (Fig. 6*A*,*B*). In contrast, only limited colocalization of endogenous BACE1, LRP1, and GM130 (a Golgi marker) was observed in soma (Fig. 6*C*). These data clearly suggest the association of endogenous BACE1 and LRP1 in endosomal compartments, particularly early endosomes, of neurons.

## Discussion

The mechanisms underlying the regulation of BACE1 in neurons have not yet been fully elucidated. It was recently shown that BACE1 is transported in axons and dendrites of neurons, where it likely functions to generate Aβ (Sannerud et al., 2011; Das et al., 2013; Deng et al., 2013; Buggia-Prévot et al., 2013; Ye and Cai, 2014). Therefore, it is critical to understand how BACE1 is regulated in axons and dendrites. BACE1 can be regulated post-translationally by modulation of intracellular trafficking and degradation (Zhi et al., 2011; Tan and Evin, 2012; Zhang and Song, 2013). It is known that BACE1 is recycled between endosomes and the TGN or the plasma membrane (Zhi et al., 2011; Tan and Evin, 2012; Zhang and Song, 2013) and cleaves APP mainly in acidic endosomal compartments (Stockley and O’Neill, 2008; Zhi et al., 2011; Tan and Evin, 2012; Zhang and Song, 2013; Vassar et al., 2014). BACE1 degradation appears to occur predominantly through the lysosomal pathway, although the involvement of the proteasomal pathway has been reported (Qing et al., 2004; Koh et al., 2005; Tesco et al., 2007, Kandalepas et al., 2013). However, the mechanisms by which BACE1 is directed to degradation pathways are poorly understood. Here, we found that LRP1 negatively regulates BACE1 through protein−protein interactions and showed that this negative regulation is mediated by modulation of BACE1 intracellular trafficking and stability.

Consistent with the finding that the expression level of BACE1 is increased in LRP1-KO cells relative to LRP1-WT cells, we observed that LRP-L4 exerts a specific suppressive effect on BACE1 in both HEK293 and primary neurons. Focusing on the mechanism of BACE1 downregulation by LRP-L4, we established that the two proteins physically interact and colocalize, and found that LRP-L4 affects the stability of BACE1, possibly through their interaction. The inhibitory effect of LRP-L4 on BACE1 expression may be independent of endocytosis. Notably, LRP-L4 influences the intracellular trafficking of BACE1; it decreases cell-surface expression of BACE1 and facilitates BACE1 transport from early to late endosomes, thereby promoting lysosomal degradation. Furthermore, we obtained evidence that endogenous BACE1 and LRP1 interact with each other and are colocalized in endosomal compartments of neurons.

**Figure 7:**
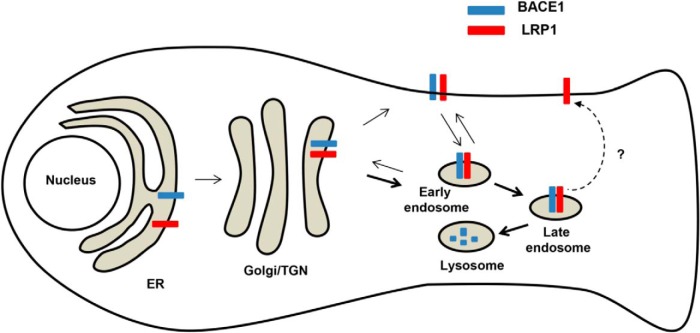
Hypothetical model. In neurons, BACE1 is recycled through the TGN, the plasma membrane and early endosomes, maintaining relative stable BACE1 levels. LRP1 complexes with BACE1 and facilitates the transit of BACE1 from early to late endosomes, promoting lysosomal targeting and degradation of BACE1. BACE1-LRP1 complexes may be sorted directly from the TGN to early endosomes. LRP1 may possibly be recycled back from late endosomes to the plasma membrane, as in the case of RAP-LRP1 complexes (Laatsch et al., 2012).

On the basis of these data, we propose the following hypothetical model (Fig. 7): BACE1 and LRP1 interact with each other and traffic from the TGN to early endosomes; this trafficking may be independent of endocytosis. BACE1−LRP1 complexes in early endosomes are preferentially sorted into late endosomes in both soma and neuronal processes of neurons. BACE1 is then degraded in lysosomes, and LRP1 may possibly be recycled back to the cell surface. Interestingly, it has been shown that RAP, one of the ligands of LRP1, is transported from early to late endosomes with LRP1 for lysosomal targeting and LRP1 is recycled back to the plasma membrane (Laatsch et al., 2012). LRP1 may thus mediate targeting of BACE1 and RAP to lysosomes by a similar trafficking mechanism. Although it is plausible that BACE1 is intimately associated with and regulated by LRP1 endogenously, additional evidence will be required to firmly establish this. Further research is also required to elucidate the molecular basis of the interaction between BACE1 and LRP1.

It has previously been shown that GGA3, one of the GGA proteins involved in the transport of cargo proteins from the TGN to endosomes, plays a significant role in the sorting of BACE1 from endosomes to lysosomes (Tesco et al., 2007). However, it is not yet clear how GGA3 participates in BACE1 regulation in neurons, especially in dendrites and axons. Another recent study also reported that snapin, a dynein motor adaptor, mediates BACE1 retrograde trafficking to lysosomes for degradation (Ye and Cai, 2014). Other factors also appear to be involved in BACE1 sorting in endocytic and recycling pathways, including sorting nexin 6, sorting nexin 12, and sortilin (He et al, 2005; Wahle et al., 2005; Okada et al., 2010; Finan et al., 2011; Zhao et al., 2012). Further research is also needed to fully understand the mechanisms by which BACE1 sorting to lysosomes is coordinately regulated by interacting proteins, including LRP1 and GGA3, in neurons.

LRP1 has previously been indicated to be one of the substrates of BACE1 (von Arnim et al., 2005; von Einem et al., 2010). However, we observed that BACE1 overexpression affects neither LRP-L4 and β-chain levels nor the intracellular expression pattern of LRP-L4. Furthermore, recent searches for BACE1 substrates in neurons using a proteomic approach did not detect LRP1 (Kuhn et al., 2012; Zhou et al., 2012). Considering these discrepant findings, LRP1 may have relatively low affinity with BACE1 as a substrate under physiological conditions.

Whether LRP1 has a role in the negative regulation of BACE1 and Aβ production *in vivo* is an important issue to be clarified. A recent study by Kanekiyo and colleagues (2013) showed that LRP1 deletion in forebrain neurons increases insoluble Aβ levels as well as amyloid burden in the cortex of APP/PS1 mice. They indicated that a disturbance of LRP1-mediated neuronal Aβ uptake and clearance is responsible for the exacerbation of Aβ deposition in neuronal LRP1-KO mice. However, their data appear to be consistent with the idea that neuronal LRP1 negatively regulates BACE1 and Aβ production, insofar as the exacerbated Aβ deposition in LRP1-KO mice may be due, at least in part, to increased neuronal Aβ production. A similar study, however, showed that lowering LRP1 levels in hippocampal neurons did not significantly alter Aβ levels and amyloid plaque numbers (Xu et al., 2012). In another study, transgenic mice expressing an LRP1 minigene exhibited small increases in the levels of both soluble and insoluble Aβ in the cortex (Zerbinatti et al., 2004). The reason for these discordant data is currently unknown.

Lipid rafts are thought to be involved in the generation and accumulation of Aβ (Vetrivel and Thinakaran 2010; Hicks et al., 2012). Previous studies have pointed to a possible significant role for exogenously expressed LRP1 C-terminal domain in the lipid raft targeting of APP as well as BACE1 (Yoon et al., 2007). However, our data using LRP1-KO cells do not support such a role for endogenous LRP1. A recent report has indicated that BACE1 cleavage of APP mainly occurs in non-raft fractions in primary neurons (Motoki et al., 2012). Whether BACE1 association with LRP1 occurs within or outside lipid rafts of endosomal compartments in neurons would be an interesting question for future research.

There have been conflicting reports on the expression levels of LRP1 protein in AD brains. Kang et al. (2000) reported reduced LRP1 expression in AD samples compared to age-matched controls; however, other studies have reported increased or unchanged LRP1 expression (Qiu et al., 2001; Causevic et al., 2003; Donahue et al., 2006). Although there does not appear to be a clear correlation between LRP1 expression and AD pathology, LRP1 plays significant roles in processes that are directly associated with the pathological progression of AD. Importantly, LRP1 expressed in brain capillaries (endothelial cells and pericytes) plays a critical role in clearance of Aβ across the blood−brain barrier (Lillis et al., 2008; Zlokovic et al., 2010; Kanekiyo and Bu, 2014). LRP1 expressed in other cell types (vascular smooth muscle cells, glial cells, and neurons) appears to mediate cellular Aβ clearance (Lillis et al., 2008; Kanekiyo and Bu, 2014). LRP1 also mediates anti-apoptotic function in neurons (Fuentealba et al., 2009) and has an essential role in synaptic function (May et al., 2004; Liu et al., 2010). Moreover, LRP1 is capable of influencing neuronal Aβ generation through BACE1 downregulation, as revealed in the current study. Thus, LRP1 has various beneficial functions that protect against AD pathology, including lowering Aβ levels in the brain. Thus, future research into LRP1 biology may aid in developing novel strategies to treat or prevent AD. For instance, any agent that is capable of upregulating LRP1 in the brain may be of therapeutic value to AD.

## Synthesis

The decision was a result of the Reviewing Editor Robert Kalb and the peer reviewers coming together and discussing their recommendations until a consensus was reached. A fact-based synthesis statement explaining their decision and outlining what is needed to prepare a revision is listed below.

Both reviewers found many merits to the work. Careful inspection of the figures reveals a number of problems that the authors need to directly address. With regard to western blot data, in figure 1E, there is clearly more APP and BACE1 in the lipid raft fractions in the KO animals (sample 4 in particularly). The conclusion that "....steady-state levels, but not lipid raft association, of BACE1 may be regulated by LRP1" is erroneous and ought to be explored more completely. Panels in figures 2 also are problematic and the authors need to address the inability to immunoblot of BACE in neurons as well as employing better controls. Both reviewers had significant issues with the immunocytochemical data. While reviewer #1 recommends deletion, this eliminate some potentially interesting observations. I encourage the authors to improve the quality of the imaging.

## References

[B1] Araki W, Yuasa K, Takeda S, Takeda K, Shirotani K, Takahashi K, Tabira T (2001) Pro-apoptotic effect of presenilin 2 (PS2) overexpression is associated with down-regulation of Bcl-2 in cultured neurons. J Neurochem 79:1161-1168. 11752057 10.1046/j.1471-4159.2001.00638.x

[B2] Araki W, Saito S, Takahashi-Sasaki N, Shiraishi H, Komano H, Murayama KS (2006) Characterization of APH-1 mutants with a disrupted transmembrane GxxxG motif. J Mol Neurosci 29:35-44. 10.1385/JMN:29:1:35 16757808

[B3] Buggia-Prévot V, Fernandez CG, Udayar V, Vetrivel KS, Elie A, Roseman J, Sasse VA, Lefkow M, Meckler X, Bhattacharyya S, George M, Kar S, Bindokas VP, Parent AT, Rajendran L, Band H, Vassar R, Thinakaran G (2013) A function for EHD family proteins in unidirectional retrograde dendritic transport of BACE1 and Alzheimer's disease Aβ production. Cell Rep 5:1552-1563. 10.1016/j.celrep.2013.12.006 24373286 PMC3932704

[B4] Cam JA, Bu G (2006) Modulation of beta-amyloid precursor protein trafficking and processing by the low density lipoprotein receptor family. Mol Neurodegener 1:8. 10.1186/1750-1326-1-8 16930455 PMC1563464

[B5] Causevic M, Ramoz N, Haroutunian V, Davis KL, Buxbaum JD (2003) Lack of association between the levels of the low-density lipoprotein receptor-related protein (LRP) and either Alzheimer dementia or LRP exon 3 genotype. J Neuropathol Exp Neurol 62:999-1005. 14575236 10.1093/jnen/62.10.999

[B6] Das U, Scott DA, Ganguly A, Koo EH, Tang Y, Roy S (2013) Activity-induced convergence of APP and BACE-1 in acidic microdomains via an endocytosis-dependent pathway. Neuron 79:447-460. 10.1016/j.neuron.2013.05.035 23931995 PMC3741682

[B7] Deng M, He W, Tan Y, Han H, Hu X, Xia K, Zhang Z, Yan R (2013) Increased expression of reticulon 3 in neurons leads to reduced axonal transport of β site amyloid precursor protein-cleaving enzyme 1. J Biol Chem 288:30236-30245. 10.1074/jbc.M113.480079 24005676 PMC3798490

[B8] Donahue JE, Flaherty SL, Johanson CE, Duncan JA 3rd, Silverberg GD, Miller MC, Tavares R, Yang W, Wu Q, Sabo E, Hovanesian V, Stopa EG (2006) RAGE, LRP-1, and amyloid-beta protein in Alzheimer's disease. Acta Neuropathol 112:405-415. 10.1007/s00401-006-0115-3 16865397

[B9] Farzan M, Schnitzler CE, Vasilieva N, Leung D, Choe H (2000) BACE2, a beta-secretase homolog, cleaves at the beta site and within the amyloid-beta region of the amyloid-beta precursor protein. Proc Natl Acad Sci U S A 97: 9712-9717. 10.1073/pnas.160115697 10931940 PMC16930

[B10] Finan GM, Okada H, Kim TW (2011) BACE1 retrograde trafficking is uniquely regulated by the cytoplasmic domain of sortilin. J Biol Chem 286:12602-12616. 10.1074/jbc.M110.170217 21245145 PMC3069461

[B11] Fuentealba RA, Liu Q, Kanekiyo T, Zhang J, Bu G (2009) Low density lipoprotein receptor-related protein 1 promotes anti-apoptotic signaling in neurons by activating Akt survival pathway. J Biol Chem 284:34045-34053. 10.1074/jbc.M109.021030 19815552 PMC2797175

[B12] Hardy J, Selkoe DJ (2002) The amyloid hypothesis of Alzheimer's disease: progress and problems on the road to therapeutics. Science 297:353-356. 10.1126/science.1072994 12130773

[B13] He X, Li F, Chang WP, Tang J (2005) GGA proteins mediate the recycling pathway of memapsin 2 (BACE). J Biol Chem 280:11696-11703. 10.1074/jbc.M411296200 15615712

[B14] Hicks DA, Nalivaeva NN, Turner AJ (2012) Lipid rafts and Alzheimer's disease: protein-lipid interactions and perturbation of signaling. Front Physiol 3:189. 10.3389/fphys.2012.00189 22737128 PMC3381238

[B15] Kametani F, Tanaka K, Ishii T, Ikeda S, Kennedy HE, Allsop D (1993) Secretory form of Alzheimer amyloid precursor protein 695 in human brain lacks β/A4 amyloid immunoreactivity. Biochem Biophys Res Commun 191:392-398. 10.1006/bbrc.1993.1230 8460999

[B16] Kandalepas PC, Sadleir KR, Eimer WA, Zhao J, Nicholson DA, Vassar R (2013) The Alzheimer's β-secretase BACE1 localizes to normal presynaptic terminals and to dystrophic presynaptic terminals surrounding amyloid plaques. Acta Neuropathol 126:329-352. 10.1007/s00401-013-1152-3 23820808 PMC3753469

[B17] Kanekiyo T, Bu G (2014) The low-density lipoprotein receptor-related protein 1 and amyloid-β clearance in Alzheimer's disease. Front Aging Neurosci 6:93. 10.3389/fnagi.2014.00093 24904407 PMC4033011

[B18] Kanekiyo T, Cirrito JR, Liu CC, Shinohara M, Li J, Schuler DR, Shinohara M, Holtzman DM, Bu G (2013) Neuronal clearance of amyloid-β by endocytic receptor LRP1. J Neurosci 33:19276-19283. 10.1523/JNEUROSCI.3487-13.2013 24305823 PMC3850043

[B19] Kang DE, Pietrzik CU, Baum L, Chevallier N, Merriam DE, Kounnas MZ, Wagner SL, Troncoso JC, Kawas CH, Katzman R, Koo EH (2000) Modulation of amyloid beta-protein clearance and Alzheimer's disease susceptibility by the LDL receptor-related protein pathway. J Clin Invest 106:1159-1166. 10.1172/JCI11013 11067868 PMC301422

[B20] Koh YH, von Arnim CA, Hyman BT, Tanzi RE, Tesco G (2005) BACE is degraded via the lysosomal pathway. J Biol Chem 280:32499-504. 10.1074/jbc.M506199200 16033761

[B21] Kuhn PH, Koroniak K, Hogl S, Colombo A, Zeitschel U, Willem M, Volbracht C, Schepers U, Imhof A, Hoffmeister A, Haass C, Roßner S, Bräse S, Lichtenthaler SF (2012) Secretome protein enrichment identifies physiological BACE1 protease substrates in neurons. EMBO J 31:3157-3168. 10.1038/emboj.2012.173 22728825 PMC3400020

[B22] Laatsch A, Panteli M, Sornsakrin M, Hoffzimmer B, Grewal T, Heeren J (2012) Low density lipoprotein receptor-related protein 1 dependent endosomal trapping and recycling of apolipoprotein E. PLoS One 7:e29385. 10.1371/journal.pone.0029385 22238606 PMC3251589

[B23] Larson ME, Lesné SE (2012) Soluble Abeta oligomer production and toxicity. J Neurochem 120 [Suppl 1]:125-139. 10.1111/j.1471-4159.2011.07478.x PMC3254782 22121920

[B24] Lasiecka ZM, Winckler B (2011) Mechanisms of polarized membrane trafficking in neurons−focusing in on endosomes. Mol Cell Neurosci 48:278-287. 10.1016/j.mcn.2011.06.013 21762782 PMC3205304

[B25] Lillis AP, Van Duyn LB, Murphy-Ullrich JE, Strickland DK (2008) LDL receptor-related protein 1: unique tissue-specific functions revealed by selective gene knockout studies. Physiol Rev 88:887-918. 10.1152/physrev.00033.2007 18626063 PMC2744109

[B26] Liu Q, Zerbinatti CV, Zhang J, Hoe HS, Wang B, Cole SL, Herz J, Muglia L, Bu G (2007) Amyloid precursor protein regulates brain apolipoprotein E and cholesterol metabolism through lipoprotein receptor LRP1. Neuron 56:66-78. 10.1016/j.neuron.2007.08.008 17920016 PMC2045076

[B27] Liu Q, Trotter J, Zhang J, Peters MM, Cheng H, Bao J, Han X, Weeber EJ, Bu G (2010) Neuronal LRP1 knockout in adult mice leads to impaired brain lipid metabolism and progressive, age-dependent synapse loss and neurodegeneration. J Neurosci 30:17068-17078. 10.1523/JNEUROSCI.4067-10.2010 21159977 PMC3146802

[B28] May P, Rohlmann A, Bock HH, Zurhove K, Marth JD, Schomburg ED, Noebels JL, Beffert U, Sweatt JD, Weeber EJ, Herz J (2004) Neuronal LRP1 functionally associates with postsynaptic proteins and is required for normal motor function in mice. Mol Cell Biol 24:8872-8883. 10.1128/MCB.24.20.8872-8883.2004 15456862 PMC517900

[B29] McNiven MA, Cao H, Pitts KR, Yoon Y (2000) The dynamin family of mechanoenzymes: pinching in new places. Trends Biochem Sci 25:115-120. 10694881 10.1016/s0968-0004(99)01538-8

[B30] Motoki K, Kume H, Oda A, Tamaoka A, Hosaka A, Kametani F, Araki W (2012) Neuronal β-amyloid generation is independent of lipid raft association of β-secretase BACE1: analysis with a palmitoylation-deficient mutant. Brain Behav 2:270-282. 10.1002/brb3.52 22741101 PMC3381632

[B31] Murayama KS, Kametani F, Araki W (2005) Extracellular release of BACE1 holoproteins from human neuronal cells. Biochem Biophys Res Commun 338:800-807. 10.1016/j.bbrc.2005.10.015 16243299

[B32] Murayama KS, Kametani F, Saito S, Kume H, Akiyama H, Araki W (2006) Reticulons RTN3 and RTN4-B/C interact with BACE1 and inhibit its ability to produce beta-amyloid protein. Eur J Neurosci 24:1237-1244. 10.1111/j.1460-9568.2006.05005.x 16965550

[B33] Okada H, Zhang W, Peterhoff C, Hwang JC, Nixon RA, Ryu SH, Kim TW (2010) Proteomic identification of sorting nexin 6 as a negative regulator of BACE1-mediated APP processing. FASEB J 24:2783-2794. 10.1096/fj.09-146357 20354142 PMC2909280

[B34] Pietrzik CU, Busse T, Merriam DE, Weggen S, Koo EH (2002) The cytoplasmic domain of the LDL receptor-related protein regulates multiple steps in APP processing. EMBO J 21:5691-5700. 12411487 10.1093/emboj/cdf568 PMC131065

[B35] Qing H, Zhou W, Christensen MA, Sun X, Tong Y, Song W (2004) Degradation of BACE by the ubiquitin-proteasome pathway. FASEB J 18:1571-1573. 10.1096/fj.04-1994fje 15289451

[B36] Qiu Z, Strickland DK, Hyman BT, Rebeck GW (2001) Elevation of LDL receptor-related protein levels via ligand interactions in Alzheimer disease and in vitro. J Neuropathol Exp Neurol 60:430-440. 11379818 10.1093/jnen/60.5.430

[B37] Rossner S, Sastre M, Bourne K, Lichtenthaler SF (2006) Transcriptional and translational regulation of BACE1 expression–implications for Alzheimer's disease. Prog Neurobiol 79:95-111. 10.1016/j.pneurobio.2006.06.001 16904810

[B38] Sannerud R, Declerck I, Peric A, Raemaekers T, Menendez G, Zhou L, Veerle B, Coen K, Munck S, De Strooper B, Schiavo G, Annaert W (2011) ADP ribosylation factor 6 (ARF6) controls amyloid precursor protein (APP) processing by mediating the endosomal sorting of BACE1. Proc Natl Acad Sci U S A 108:E559-568. 10.1073/pnas.1100745108 21825135 PMC3161548

[B39] Stockley JH, O'Neill C (2008) Understanding BACE1: essential protease for amyloid-beta production in Alzheimer's disease. Cell Mol Life Sci 65:3265-3289. 10.1007/s00018-008-8271-3 18695942 PMC11131673

[B40] Sun X, Bromley-Brits K, Song W (2012) Regulation of β-site APP-cleaving enzyme 1 gene expression and its role in Alzheimer's disease. J Neurochem 120 Suppl 1:62-70. 10.1111/j.1471-4159.2011.07515.x 22122349

[B41] Takeda K, Araki W, Akiyama H, Tabira T (2004) Amino-truncated amyloid β-peptide (Aβ5-40/42) produced from caspase-cleaved amyloid precursor protein is deposited in Alzheimer's disease brain. FASEB J 18:1755-1757. 10.1096/fj.03-1070fje 15364896

[B42] Takeda T, Yamazaki H, Farquhar MG (2003) Identification of an apical sorting determinant in the cytoplasmic tail of megalin. Am J Physiol Cell Physiol 284:C1105-1113. 10.1152/ajpcell.00514.2002 12519751

[B43] Tan J, Evin G (2012) Β-site APP-cleaving enzyme 1 trafficking and Alzheimer's disease pathogenesis. J Neurochem 120:869-880. 10.1111/j.1471-4159.2011.07623.x 22171895

[B44] Tesco G, Koh YH, Kang EL, Cameron AN, Das S, Sena-Esteves M, Hiltunen M, Yang SH, Zhong Z, Shen Y, Simpkins JW, Tanzi RE (2007) Depletion of GGA3 stabilizes BACE and enhances beta-secretase activity. Neuron 54:721-737. 10.1016/j.neuron.2007.05.012 17553422 PMC1973166

[B45] Ulery PG, Strickland GK (2000) LRP in Alzheimer’s disease. J Clin Invest 106: 1077-1079. 10.1172/JCI11455 11067860 PMC301424

[B46] Vassar R, Bennett BD, Babu-Khan S, Kahn S, Mendiaz EA, Denis P, Teplow DB, Ross S, Amarante P, Loeloff R, Luo Y, Fisher S, Fuller J, Edenson S, Lile J, Jarosinski MA, Biere AL, Curran E, Burgess T, Louis JC, Collins F, Treanor J, Rogers G, Citron M (1999) Beta-secretase cleavage of Alzheimer's amyloid precursor protein by the transmembrane aspartic protease BACE. Science 286:735-741. 10531052 10.1126/science.286.5440.735

[B47] Vassar R, Kuhn PH, Haass C, Kennedy ME, Rajendran L, Wong PC, Lichtenthaler SF (2014) Function, therapeutic potential and cell biology of BACE proteases: current status and future prospects. J Neurochem 130:4-28. 10.1111/jnc.12715 24646365 PMC4086641

[B48] Vetrivel KS, Thinakaran G (2010) Membrane rafts in Alzheimer's disease β-amyloid production. Biochim Biophys Acta 1801:860-867. 10.1016/j.bbalip.2010.03.007 20303415 PMC2886169

[B49] von Arnim CA, Kinoshita A, Peltan ID, Tangredi MM, Herl L, Lee BM, Spoelgen R, Hshieh TT, Ranganathan S, Battey FD, Liu CX, Bacskai BJ, Sever S, Irizarry MC, Strickland DK, Hyman BT (2005) The low density lipoprotein receptor-related protein (LRP) is a novel beta-secretase (BACE1) substrate. J Biol Chem 280:17777-17785. 10.1074/jbc.M414248200 15749709

[B50] von Einem B, Schwanzar D, Rehn F, Beyer AS, Weber P, Wagner M, Schneckenburger H, von Arnim CA (2010) The role of low-density receptor-related protein 1 (LRP1) as a competitive substrate of the amyloid precursor protein (APP) for BACE1. Exp Neurol 225:85-93. 10.1016/j.expneurol.2010.05.017 20685197

[B51] Wagner T, Pietrzik CU (2012) The role of lipoprotein receptors on the physiological function of APP. Exp Brain Res 217:377-387. 10.1007/s00221-011-2876-8 21947084

[B52] Wahle T, Prager K, Raffler N, Haass C, Famulok M, Walter J (2005) GGA proteins regulate retrograde transport of BACE1 from endosomes to the trans-Golgi network. Mol Cell Neurosci 29:453-461. 10.1016/j.mcn.2005.03.014 15886016

[B53] Xu G, Green CC, Fromholt SE, Borchelt DR (2012) Reduction of low-density lipoprotein receptor-related protein (LRP1) in hippocampal neurons does not proportionately reduce, or otherwise alter, amyloid deposition in APPswe/PS1dE9 transgenic mice. Alzheimers Res Ther 4:12. 10.1186/alzrt110 22537779 PMC4054673

[B54] Ye X, Cai Q (2014) Snapin-mediated BACE1 retrograde transport is essential for its degradation in lysosomes and regulation of APP processing in neurons. Cell Rep 6:24-31. 10.1016/j.celrep.2013.12.008 24373968 PMC3905048

[B55] Yoon IS, Chen E, Busse T, Repetto E, Lakshmana MK, Koo EH, Kang DE (2007) Low-density lipoprotein receptor-related protein promotes amyloid precursor protein trafficking to lipid rafts in the endocytic pathway. FASEB J 21:2742-2752. 10.1096/fj.07-8114com 17463224

[B56] Zerbinatti CV, Wozniak DF, Cirrito J, Cam JA, Osaka H, Bales KR, Zhuo M, Paul SM, Holtzman DM, Bu G (2004) Increased soluble amyloid-beta peptide and memory deficits in amyloid model mice overexpressing the low-density lipoprotein receptor-related protein. Proc Natl Acad Sci U S A 101:1075-1080. 10.1073/pnas.0305803101 14732699 PMC327153

[B57] Zhang X, Song W (2013) The role of APP and BACE1 trafficking in APP processing and amyloid-β generation. Alzheimers Res Ther 5:46. 10.1186/alzrt211 24103387 PMC3978418

[B58] Zhao J, Fu Y, Yasvoina M, Shao P, Hitt B, O'Connor T, Logan S, Maus E, Citron M, Berry R, Binder L, Vassar R (2007) Beta-site amyloid precursor protein cleaving enzyme 1 levels become elevated in neurons around amyloid plaques: implications for Alzheimer's disease pathogenesis. J Neurosci 27:3639-3649. 10.1523/JNEUROSCI.4396-06.2007 17409228 PMC6672403

[B59] Zhao Y, Wang Y, Yang J, Wang X, Zhao Y, Zhang X, Zhang YW (2012) Sorting nexin 12 interacts with BACE1 and regulates BACE1-mediated APP processing. Mol Neurodegener 7:30. 10.1186/1750-1326-7-30 22709416 PMC3439308

[B60] Zhi P, Chia PZ, Gleeson PA (2011) Intracellular trafficking of the β-secretase and processing of amyloid precursor protein. IUBMB Life 63:721-729. 10.1002/iub.512 21834057

[B61] Zhou L, Brouwers N, Benilova I, Vandersteen A, Mercken M, Van Laere K, Van Damme P, Demedts D, Van Leuven F, Sleegers K, Broersen K, Van Broeckhoven C, Vandenberghe R, De Strooper B (2011) Amyloid precursor protein mutation E682K at the alternative β-secretase cleavage β'-site increases Aβ generation. EMBO Mol Med 3:291-302. 10.1002/emmm.201100138 21500352 PMC3377078

[B62] Zhou L, Barão S, Laga M, Bockstael K, Borgers M, Gijsen H, Annaert W, Moechars D, Mercken M, Gevaert K, De Strooper B (2012) The neural cell adhesion molecules L1 and CHL1 are cleaved by BACE1 protease in vivo. J Biol Chem 287:25927-25940. 10.1074/jbc.M112.377465 22692213 PMC3406677

[B63] Zlokovic BV, Deane R, Sagare AP, Bell RD, Winkler EA (2010) Low-density lipoprotein receptor-related protein-1: a serial clearance homeostatic mechanism controlling Alzheimer’s amyloid β-peptide elimination from the brain. J Neurochem 115:1077-1089. 10.1111/j.1471-4159.2010.07002.x 20854368 PMC2972355

